# ﻿*Lysimachiaailaoshanensis* (Primulaceae), a new species from Yunnan, China

**DOI:** 10.3897/phytokeys.246.130838

**Published:** 2024-09-17

**Authors:** Hai-Fei Yan, Gang Hao

**Affiliations:** 1 State Key Laboratory of Plant Diversity and Specialty Crops, South China Botanical Garden, Chinese Academy of Sciences, Guangzhou 510650, China; 2 South China National Botanical Garden, Guangzhou 510650, China; 3 College of Life Sciences, South China Agricultural University, Guangzhou 510642, Guangdong, China

**Keywords:** Ericales, flora, morphological features, taxonomy, Yunnan

## Abstract

A new species, *Lysimachiaailaoshanensis* is described and illustrated. In gross morphology it is evidently allied to subgen. Palladiasect.Chenopodiopsis and is most similar to *L.chenopodioides* and *L.remotiflora*, but is distinguished from *L.chenopodioides* by narrower lanceolate leaf blade and longer pedicel, and longer stamens and styles, and from *L.remotiflora* by narrower leaf blade and longer stamens.

## ﻿Introduction

Ailao Mountain, located in central Yunnan, China, has rich plant diversity and was designated as a national natural reserve in 1988. In August 2020, Dr. H.F. Yan and colleagues of South China Botanical Garden made a botanical excursion to Ailao Mt., collecting plants of this area for systematic study on Primulaceae. A putatively new species of *Lysimachia* was secured. A subsequent field trip was conducted to confirm its entity. Careful examination revealed that the plant is distinct from all other *Lysimachia* species and represents an undescribed taxon.

## ﻿Materials and methods

Earlier taxonomic literature has been consulted (e.g. Handel-Mazzetti 1928; Chen and Hu 1979; Chen et al. 1989; Hu 1985, 1992, 1999; Hu and Kelso 1996) to infer allied species and relatedness. The new species was examined in the field and at the herbarium, and measurements of morphological features were conducted with fresh specimens. Flowers were dissected and measured in the laboratory. Morphological comparison with similar species was performed based on living plants and specimens from IBSC, KUN, PE, IBK and from the images of specimens from the JSTOR Global Plants (http://plants.jstor.org/). The conservation status of the new species was assessed following the guidelines for using the IUCN Red List Categories and Criteria (IUCN Standards and Petitions Committee 2024).

## ﻿Taxonomic treatment

### ﻿ Lysimachia ailaoshanensis

Taxon classificationPlantaeEricalesPrimulaceae

G.Hao & Y.F.Yan sp. nov.

962ED6FA-70A8-5BDF-80FB-37845FEFC41B

urn:lsid:ipni.org:names:77348638-1

Figs 1 , 2

#### Type.

China. • Yunnan Province, Jingdong Yi Autonomous County, Xujiaba, near Damenkou; 24°31'N, 101°00'E; alt. 2363 m; 14 Aug. 2020; *Hai-Fei Yan et al. Y2020286* (holotype: IBSC! barcode IBSC1021506; isotypes: IBSC! barcode IBSC1025535, IBSC1025536).

#### Diagnosis.

*Lysimachiaailaoshanensis* is most similar to *L.chenopodioides* Watt ex Hook. f. and *L.remotiflora* C.M. Hu, but differs from *L.chenopodioides* in narrower lanceolate leaf blade and longer pedicel, and longer stamens and styles, and from *L.remotiflora* in narrower leaf blade and longer stamens.

#### Description.

Herbs annual, glabrous, 18 to 58 cm tall. Stems erect to ascending-erect, quadrangular, branches usually few above middle. Leaves alternate; petiole 1–2.8 cm long, narrowly winged; leaf blade narrowly lanceolate, 1.8–6.0 × 0.5–1.5 cm, sparsely dark purple or brown glandular punctate, base attenuate, apex acuminate to acute. Pedicel 0.5–2 cm long. Flowers in axils of upper leaves, always forming a raceme of 5–18 cm, lax. Calyx lobes lanceolate, 4.5–5.5 mm long, split nearly to base, dark purple or black glandular striate outside, apex obtuse to subacute. Corolla white or pink; tube ca. 1 mm long; lobes oblong-spatulate, 4.5–5 mm long, dark purple glandular striate, apex obtuse. Stamens ca. as long as to slightly shorter than corolla lobes; filaments adnate to base of corolla lobes, free parts ca. 4.0 mm; anthers ovate, dorsifixed, ca. 0.5 mm. Ovary glabrous; style ca. 4.5 mm. Capsule globose, ca. 4 mm in diameter, glabrous.

#### Distribution and habitat.

**Map 1.**
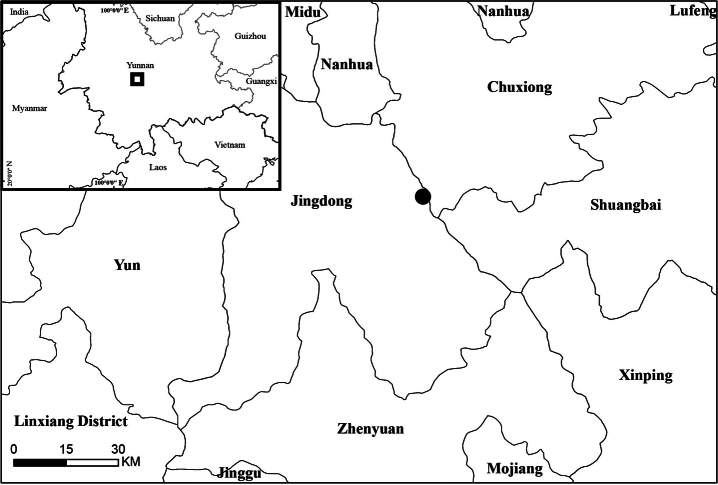
Location of the population of *Lysimachiaailaoshanensis* in Jingdong, Yunnan.

The new species is presently known only from the type locality in Yunnan Province, Jingdong Yi Autonomous County (Map 1). It grows at the edge of secondary mixed-evergreen forests.

#### Phenology.

Flowering from June to August, fruiting from July to August.

#### Etymology.

The new species is named referring to the type locality where the new species occurs, Ailaoshan National Nature Reserve, Yunnan, China.

#### Conservation status.

Based on our field investigations in Jingdong Yi Autonomous County and adjacent areas in the past three years, only one population with only five individuals of the new species have been found in an area of 10 km^2^ in Jingdong Yi Autonomous County. Moreover, the local habitat is under threat by road construction and tourism development. Therefore, the conservation status of the new species is assessed as Critically Endangered (CR) (B2a & bi, iii), according to the guidelines for using the IUCN Red List Categories and Criteria (IUCN Standards and Petitions Committee 2024).

#### Additional specimens examined (paratype).

China. The same locality as holotype, 7 August 2023, *Hai-Fei Yan Yan2023054* (IBSC! barcode IBSC1025537, IBSC1025538).

#### Relationship with similar species.

**Figure 1.**
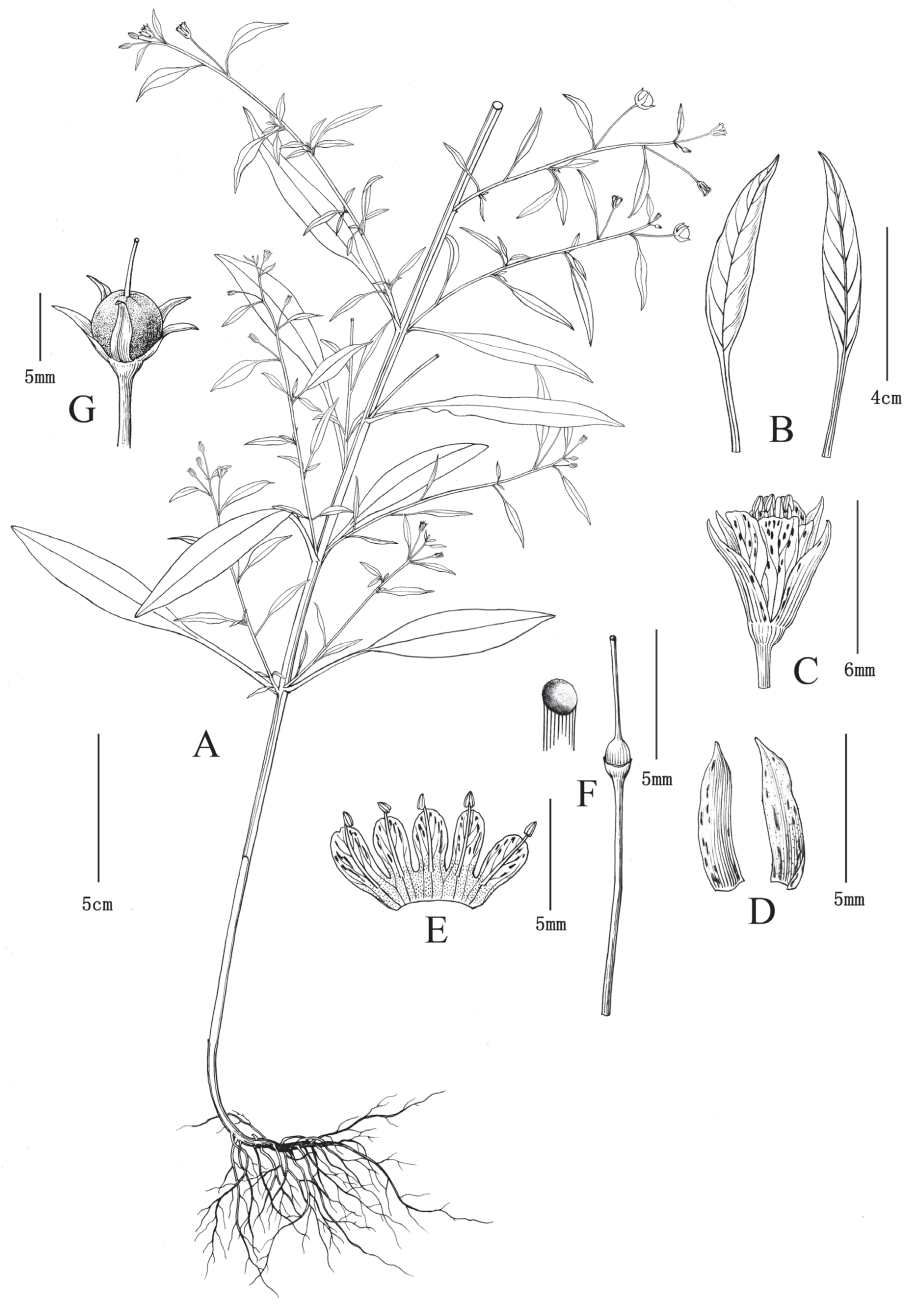
*Lysimachiaailaoshanensis* G.Hao & Y.F.Yan, sp. nov. **A** habit **B** abaxial (right) and adaxial (left) surfaces of a leaf **C** flower **D** calyx lobes **E** dissected corolla **F** pistil and its stigma (enlarged) **G** young fruit with persistent calyx. Drawn by Yun-Xiao Liu from the holotype.

**Figure 2.**
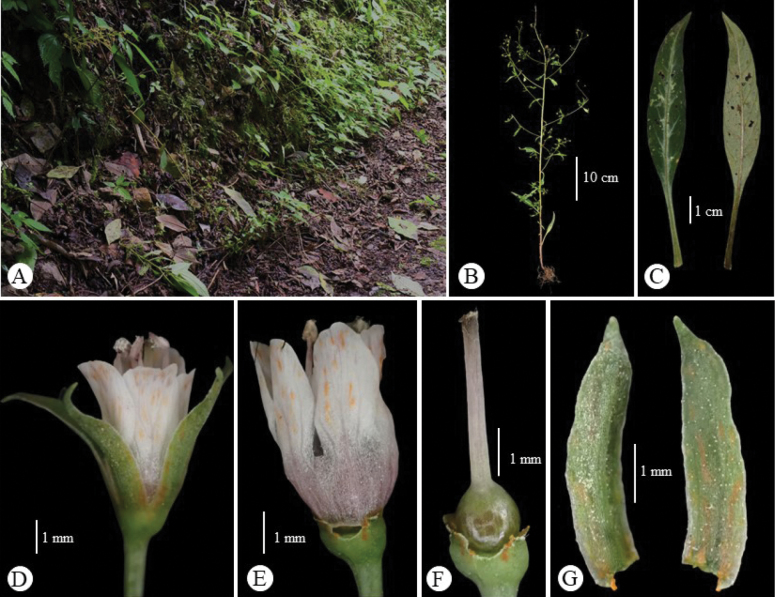
Living plant of *Lysimachiaailaoshanensis* G.Hao & Y.F.Yan, sp. nov. **A** habitat **B** habit **C** leaves on abaxial (right) and adaxial (left) surfaces **D** flower (lateral view) **E** corolla **F** pistil **G** abaxial (left) and adaxial (right) sides of a calyx lobe. Photographed by Hai-Fei Yan.

**Figure 3.**
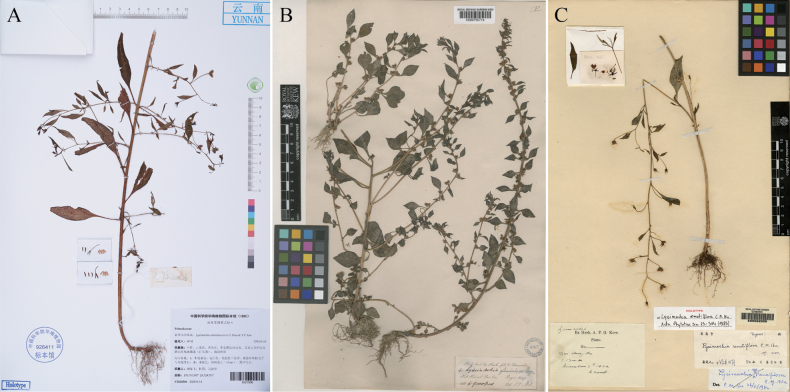
Holotypes of *Lysimachiaailaoshanensis* and two of its allies **A***L.ailaoshanensis***B***L.chenopodioides***C***L.remotiflora*.

Based on the classification of *Lysimachia* by Handel-Mazzetti (1928) and Chen and Hu (1979), the new species clearly belongs to Lysimachiasubg.Palladiasect.Chenopodiopsis Hand.-Mazz., which is characterised by leaves alternate, racemes sparsely flowered or solitary in axils of upper leaves, filaments free, adnate to middle of corolla, and styles usually shorter than corolla. Approximately eight species were recognized in this section, mainly distributed in southwestern China and adjacent regions (e.g., Bhutan, India, Kashmir, N. Myanmar, Nepal, Pakistan), and a few outliers in Thailand, the Mediterranean coast, and southeastern Africa (Handel-Mazzetti 1928; Chen et al. 1989; Hu and Kelso 1996). The new species is morphologically similar to *L.chenopodioides* and *L.remotiflora*, but is distinctive in its leaf shape and heights of stamens and styles (see Table 1, Figs 1–3).

**Table 1.** Main morphological differences between *Lysimachiaailaoshanensis* and two similar species.

Features	*L.ailaoshanensis*	*L.chenopodioides*	*L.remotiflora*
Petiole length	1–2.8 cm	0.5–1 cm	ca. 1.1 cm
Lamina shape	narrowly lanceolate	ovate to rhomboid-ovate	ovate-lanceolate
Pedicel length	0.5–2 cm	1–2 mm	1.5–2.5 cm
Filament length	4.5–5 mm	1–1.5 mm	1–1.5 mm
Style length	4.5 mm	1.5 mm	2.5 mm

## Supplementary Material

XML Treatment for Lysimachia ailaoshanensis

## ﻿Additional information

### Data availability

All of the data that support the findings of this study are available in the main text.
